# Review of Chinese Oligaphorurini (Collembola, Onychiuridae) with descriptions of two new Palaearctic species

**DOI:** 10.3897/zookeys.192.2959

**Published:** 2012-05-08

**Authors:** Xin Sun, Donghui Wu

**Affiliations:** 1 Key laboratory of Wetland Ecology and Environment, Northeast Institute of Geography and Agroecology, Chinese Academy of Sciences, Changchun 130012, China; 2 College of Earth Science, Jilin University, Changchun 130061, China

**Keywords:** Collembola, identification key, *Micraphorura changbaiensis* sp. n., *Oligaphorura pseudomontana* sp. n., taxonomy

## Abstract

A checklist of Chinese Oligaphorurini is given. Two new Chinese species, *Micraphorura changbaiensis* **sp. n.** and *Oligaphorura pseudomontana* **sp. n.**, are described from Changbai Mountain Range. *Micraphorura changbaiensis* sp. n. has the same dorsal pseudocelli formula and number of papillae in Ant. III sensory organ as *Micraphorura uralica*, but they can be easily distinguished by number of chaetae in Ant. III sensory organ, ventral pseudocelli formula, ventral parapseudocelli formula, number of pseudocelli on subcoxa 1 of legs I–III, dorsal axial chaeta on Abd. V and number of chaetae on tibiotarsi. *Oligaphorura pseudomontana* sp. n. is very similar to the species *Oligaphorura montana* having an increased number of pseudocelli on body dorsally, well marked base of antenna with 1 pseudocellus and 3 pseudocelli outside, subcoxa 1 of legs I–III with 1 pseudocellus each, dorsally S-chaetae formula as 11/011/22211 from head to Abd. V, S-microchaeta present on Th. II–III, claw without inner teeth and with 1+1 lateral teeth, and unguiculus with basal lamella; but they can be separated easily by the number of pseudocelli on Abd. V and VI terga, parapseudocelli on the body, number of chaetae on Th. I tergum, and number of chaetae on tibiotarsi. A key to Chinese species of Oligaphorurini is provided in the present paper.

## Introduction

The tribe Oligaphorurini, erected by Bagnall (1949) as a subfamily, is characterized by having a small postantennal organ with a 3–5 lobed vesicle. So far, 38 species belonging to five genera were reported in the world (Bellinger et al. 2012). Nevertheless, the Chinese fauna of Oligaphorurini is poorly known, only two species, *Dimorphaphorura sanjiangensis* Sun & Wu, 2012 and *Oligaphorura ursi* (Fjellberg, 1984), were reported from northeast China (Sun and Wu 2012).

In the present paper, two new Chinese Oligaphorurini species are described from Changbai Mountain Range in Jilin Province, and two newly recorded species, *Oligaphorura judithae* (Weiner, 1994) and *Oligaphorura koreana* (Weiner, 1994), are mentioned. A checklist of Chinese Oligaphorurini and an identification key to all Chinese species of this tribe are given below.

## Material and methods

Specimens were mounted in Marc André II solution, after clearing in lactic acid, and were studied using a Nikon Eclipse 80i microscope. Material is deposited in the Key laboratory of Wetland Ecology and Environment, Northeast Institute of Geography and Agroecology, Chinese Academy of Sciences, Changchun.

### Abbreviations used in descriptions

Labial papillae types are named after Fjellberg (1999). Labium areas and chaetal nomenclature follow Massoud (1967) and D’Haese (2003). Chaetae on anal valves are named after Yoshii (1996).

Ant.–antennal segments, PAO–postantennal organ, Th.–thoracic segments, Abd.–abdominal segments, p-chaeta–chaeta of row p, Sp–posterior S-chaeta (e.g. on Abd. V or on head), ms–S-microchaeta, pso–pseudocellus, a-pso–postero-internal pso on head, psx–parapseudocellus, psp–pseudopore, x–axial pseudopore of Abd. IV.

Pseudocelli, parapseudocelli and pseudopore formula are the number of pseudocelli, parapseudocelli or pseudopores by half tergum (dorsally) or half-sternum (ventrally) as follows: head anterior, head posterior/Th. I, Th. II, Th. III/Abd. I, Abd. II, Abd. III, Abd. IV, Abd. V (for instance: 43/144/54464).

S-chaetae formula is the number of S-chaetae by half tergum from head to Abd. VI (for instance: 11/011/222111).

Tibiotarsus chaetotaxy formula: total number of chaetae (number of basal chaetae, number of chaetae in row B, number of chaetae in distal row A+T, for instance: 19 (1, 7, 11).

### Checklist of Chinese species of Oligaphorurini Bagnall, 1949

***Dimorphaphorura sanjiangensis*** Sun & Wu, 2012

**Distribution.** Heilongjiang Province (according to the original paper), Jilin Province (Changbai Mountain Range, alt. 689m, 43.037640°N, 128.199653°E).

***Micraphorura changbaiensis* sp. n.**

**Distribution.** Jilin Province.

***Oligaphorura judithae* (Weiner, 1994) newly recorded in China**

**Distribution.** Jilin Province (Changbai Mountain Range, alt. 689m, 43.037640°N, 128.199653°E); North Korea (according to the original paper).

***Oligaphorura koreana* (Weiner, 1994) newly recorded in China**

**Distribution.** Jilin Province (Changbai Mountain Range, alt. 689m, 43.037640°N, 128.199653°E and alt. 1763m, 41.755265°N, 127.941123°E); North Korea (according to the original paper).

***Oligaphorura ursi* (Fjellberg, 1984)**

**Distribution.** Jilin Province (Changbai Mountain Range, alt. 689m, 43.037640°N, 128.199653°E), Heilongjiang Province (according to Sun and Wu 2012); Northern Holarctic (according to the original paper).

***Oligaphorura pseudomontana* sp. n.**

**Distribution.** Jilin Province.

### Key to Chinese species of Oligaphorurini Bagnall, 1949

**Table:** 

1	Furca reduced to a finely granulated area, with 1+1 dental chaetae posteriorly (*Dimorphaphorura* Bagnall, 1949)	*Dimorphaphorura sanjiangensis* Sun & Wu, 2012
–	Furca reduced to a small cuticular fold, with 1+1 or 2+2 dental chaetae posteriorly	2
2	1+1 dental chaetae posteriorly (*Micraphorura* Bagnall, 1949)	*Micraphorura changbaiensis* sp. n.
–	2+2 dental chaetae in two rows posteriorly (*Oligaphorura* Bagnall, 1949)	3
3	First thoracic tergum with 0+0 pso	*Oligaphorura koreana* (Weiner, 1994)
–	First thoracic tergum with 1+1 pso	4
4	The base of antenna with 4+4 pso	*Oligaphorura pseudomontana* sp. n.
–	The base of antenna with 3+3 pso	5
5	Lateral ms absent on Th. III, 5+5 pso on Abd. IV and 4+4 pso on Abd. V	*Oligaphorura judithae* (Weiner, 1994)
–	Lateral ms present on Th. III, 4+4 pso on Abd. IV and 3+3 pso on Abd. V	*Oligaphorura ursi* (Fjellberg, 1984)

## Systematics

### Micraphorura changbaiensis sp. n.

urn:lsid:zoobank.org:act:E9432AE4-B11B-4F86-B581-BC15BC6A85E8

http://species-id.net/wiki/Micraphorura_changbaiensis

Figs 1 –2

#### Type material.

Holotype female, 4 female and 1 male paratypes. China: Jilin Province: Changbai Mountain Range (alt. 1763m, 41.755265°N, 127.941123°E): 15.VIII.2009, litter and soil, Berlese extraction, Wu Dong-hui leg.

Holotype and 5 paratypes on slides are deposited in the Key laboratory of Wetland Ecology and Environment, Northeast Institute of Geography and Agroecology, Chinese Academy of Sciences, Changchun.

#### Diagnosis.

Pso formula as 32/133/44454 dorsally and 11/000/00000 ventrally; subcoxa 1 of legs I–III with 1 pso each; psx formula as 00/000/222200 ventrally, absent dorsally; S-chaetae formula as 11/011/222111 dorsally and 11/000/000100 ventrally; Ant. III sensory organ composed of 5 papillae, 5 guard chaetae, 2 small sensory rods, 2 granulated sensory clubs; Abd. V tergum with one dorsal axial chaeta (p0), Abd. VI with two axial chaetae (a0 and p0); tibiotarsi of legs I, II and III with 20, 20 and 19 chaetae; anal spines present on indistinct papillae, as long as inner edge of unguis.

#### Description.

Body color white in alcohol. Size 0.70–0.82 mm, holotype: 0.78 mm. Body slender and elongated.

**Figure 1.**
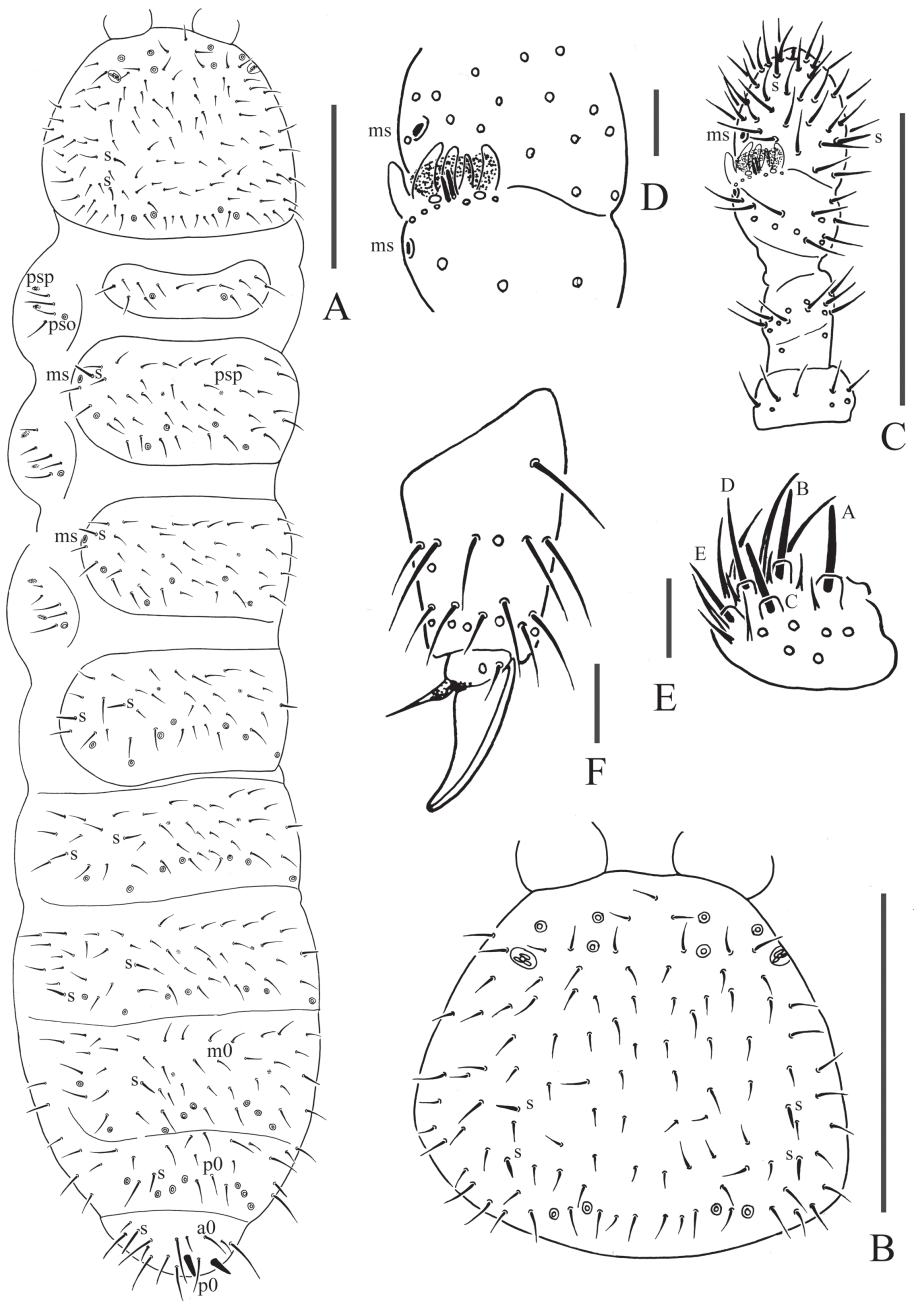
*Micraphorura changbaiensis* sp. n. **A** dorsal side of body **B** dorsal side of head **C** antenna **D** organ of Ant. III **E** labium **F** distal part of leg III. Scales: 0.1 mm (A–C), 0.01 (D–F)

**Figure 2.**
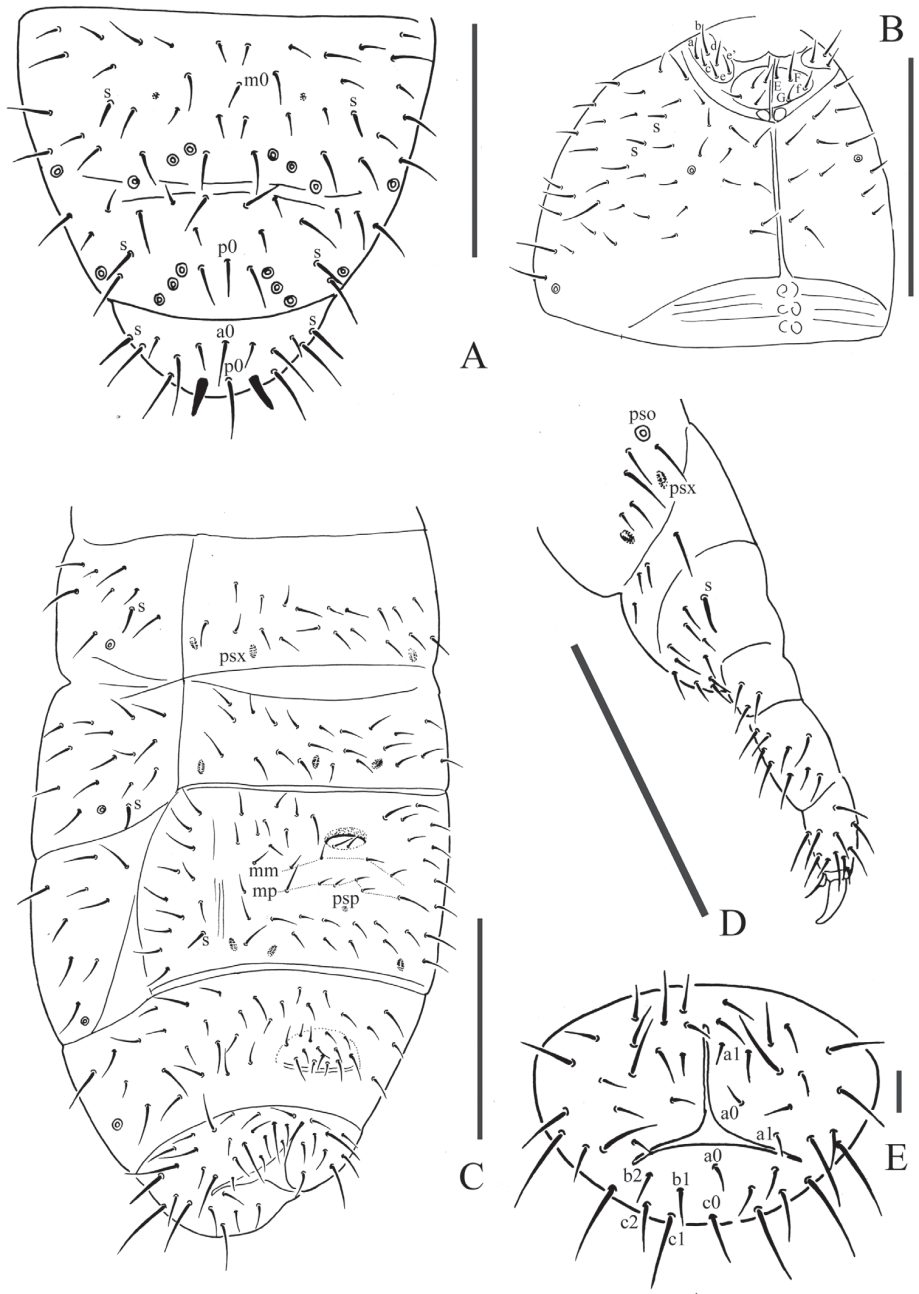
*Micraphorura changbaiensis* sp. n. **A** dorsal side of Abd. IV–VI **B** ventral side of head **C** ventral side of Abd. II–VI **D** leg III **E** anal valves. Scales: 0.1 mm (A–D), 0.01 (E)

Pseudocelli (pso) formula as 32/133/44454 dorsally and 11/000/00000 ventrally (Figs 1A, 2C); subcoxa 1 of legs I–III with 1 pso each. Parapseudocelli (psx) formula as 00/000/222200 ventrally and absent dorsally (Figs 1A, 2C); subcoxa 1 of legs I–III with 2 psx each. Pseudopore (psp) formula as 00/011/111100 dorsallyand 00/111/000x00 ventrally (Figs 1A, 2C).

S-chaetae cylindrical, well differentiated, formula as 11/011/222111 dorsallyand 11/000/000100 ventrally (Figs 1A, 2C); subcoxae 2 of legs I, II and III with 0, 0 and 1 S-chaeta respectively (Fig. 2D). Two posterior S-chaetae (Sp) present on head. S-microchaetae tiny and blunt, present on Th. II–III (Fig. 1A).

Head. Antennae short and distinctly segmented, 0.8 times as long as head. Length ratio of antennal segments I: II: III: IV = 1: 1.8: 1.8: 2. Ant. IV with two distinct thickened S-chaetae, subapical organite with apex globular and basolateral ms just above posterior chaetae (Fig. 1C). Ant. III sensory organ composed of 5 papillae, 5 guard chaetae, 2 small sensory rods, 2 granulated sensory clubs, the outer about twice as large as the inner, and a lateral ms (Fig. 1D). Ant. II with 14 chaetae. Ant. I with 8 chaetae. Antennal base without distinct granulation. PAO located on cuticular furrow built with a 3–4 lobed vesicle (Fig. 1B). Dorsal cephalic chaeta d0 absent, 3+3 p-chaetae between posterior a-pso on head (Fig. 1B). Mandible with strong molar plate and 4 apical teeth. Maxilla bearing 3 teeth and 6 lamellae. Maxillary palp simple with 1 basal chaeta and 2 sublobal hairs. Labral chaetae formula 4/342. Labium with 6 proximal, 4 basomedian (E, F, G, f) and 6 basolateral (a, b, c, d, e, e’) chaetae (Fig. 2B); labial type ABC, papillae A–E respectively with 1, 4, 0, 3, 3 guard chaetae (Fig. 1E). Postlabial chaetae 4+4 along ventral groove (Fig. 2B).

Body chaetotaxy. Ordinary chaetae differentiated in meso- and macrochaetae. Th. I tergum with 7+7 chaetae dorsally (Fig. 1A). Th. II–III and Abd. I–III terga with three chaetae on both side of axial line and no dorsal axial chaetae. Abd. IV tergum with one dorsal axial chaeta (m0), Abd. V with one dorsal axial chaeta (p0), Abd. VI with two axial chaetae (a0 and p0) (Fig. 2A). Th. I, II and III sterna with 0+0/1+1/1+1 (2+2) chaetae.

Appendages. Subcoxa 1 of legs I–III with 4, 5 and 5 chaetae, subcoxa 2 with 1, 4 and 4 chaetae respectively. Tibiotarsi of legs I, II and III with 20 (1, 8, 11), 20 (1, 8, 11) and 19 (1, 7, 11) chaetae (Fig. 1F). Unguis without teeth. Unguiculus slender and pointed, 0.6 times as long as inner edge of unguis, with inner basal lamella (Figs 1F, 2D). Ventral tube with 6+6 distal chaetae and 2+2 basal chaetae, without anterior chaetae. Furca reduced to a small cuticular fold with 1+1 dental chaetae posteriorly; two manubrial rows of chaetae present (Fig. 2C).

Female genital plate with 14 chaetae; in our specimens, the only male is juvenile. Anal valves with numerous acuminate chaetae; each lateral valve with a0 and 2a1; upper valves with chaetae a0, 2b1, 2b2, c0, 2c1, 2c2 (Fig. 2E). Anal spines present on indistinct papillae, as long as inner edge of unguis (Fig. 1A).

#### Etymology.

Named after the mountain range where we found the new species.

#### Ecology.

Found in coniferous forest.

#### Remarks.

The new species has the same dorsal pseudocelli formula (32/133/44454) and number of papillae in Ant. III sensory organ (5) as *Micraphorura uralica* (Khanislamova, 1986), but they can be easily distinguished by number of guard chaetae on Ant. III sensory organ (5 in *changbaiensis* sp. n., 4 in *uralica*), ventral pseudocelli formulae (11/000/00000 in *changbaiensis* sp. n., 11/000/11120 in *uralica*), ventral parapseudocelli formulae (00/000/222200 in *changbaiensis* sp. n., indistinct in *uralica*), pseudocelli on subcoxa 1 of legs I–III (1, 1, 1 in *changbaiensis* sp. n., 2, 3, 3 in *uralica*), dorsal axial chaeta on Abd. V (m0 in *changbaiensis* sp. n., absent in *uralica*), and number of chaetae on tibiotarsi (20, 20, 19 in *changbaiensis* sp. n., 19, 19, 18 in *uralica*).

### Oligaphorura pseudomontana sp. n.

urn:lsid:zoobank.org:act:6691C23D-A275-4C91-B001-C4C5AFCC8D04

http://species-id.net/wiki/Oligaphorura_pseudomontana

Figs 3 –4

#### Type material.

Holotype male, 2 female and 5 male paratypes. China: Jilin Province: Changbai Mountain Range (alt. 689m, 43.037640°N, 128.199653°E): 3.X.2011, litter and soil, Berlese extraction, Tang Xu-guang leg.

Holotype and 7 paratypes on slides are deposited in the Key laboratory of Wetland Ecology and Environment, Northeast Institute of Geography and Agroecology, Chinese Academy of Sciences, Changchun.

#### Diagnosis.

Pso formula as 43/144/54464 dorsally and 11/000/00000 ventrally; subcoxa 1 of legs I–III with 1 pso each; psx formula as 00/000/222401 ventrally and absent dorsally; S-chaetae formula as 11/011/222111 dorsally and 11/000/000100 ventrally; Ant. III sensory organ composed of 5 papillae, 5 guard chaetae, 2 small sensory rods, 2 granulated sensory clubs; Abd. V without dorsal axial chaetae, Abd. VI with two axial chaetae (a0 and p0); tibiotarsi of legs I, II and III with 20, 20 and 19 chaetae; anal spines present on indistinct papillae, 0.75 times as long as inner edge of unguis.

#### Description.

Body color white in alcohol. Size 0.80–1.04 mm; holotype: 0.90 mm. Body slender and elongated.

**Figure 3.**
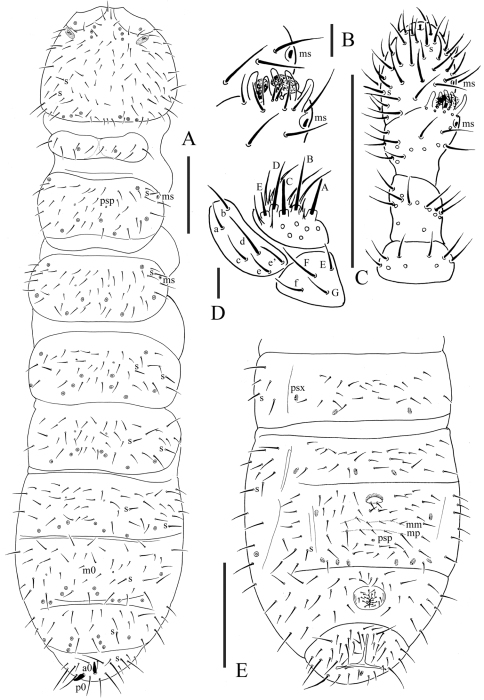
*Oligaphorura pseudomontana* sp. n. **A** dorsal side of body **B** organ of Ant. III **C** antenna **D** labium **E** ventral side of Abd. II–VI. Scales: 0.1 mm (A, C & E), 0.01 (B & D)

**Figure 4.**
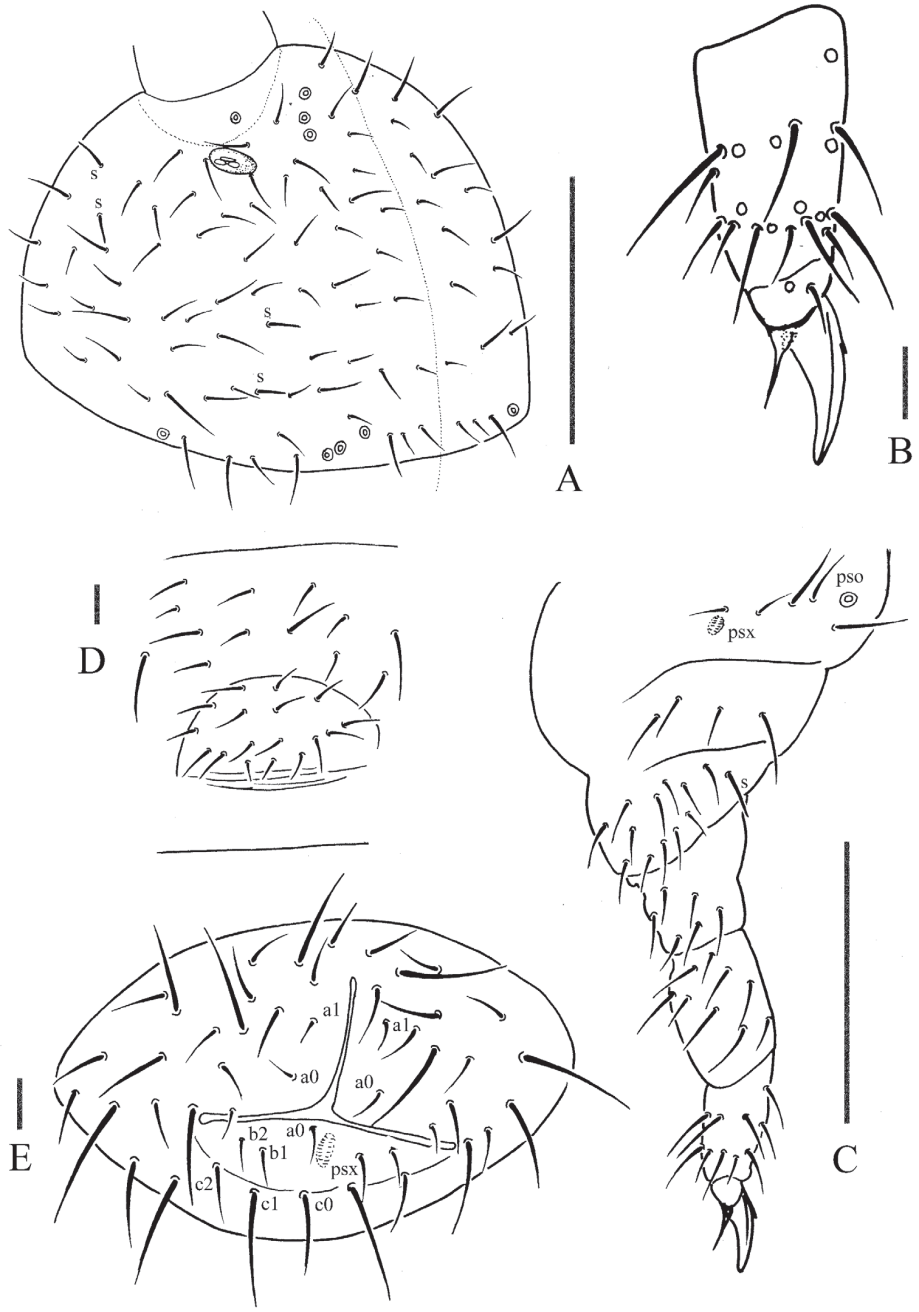
*Oligaphorura pseudomontana* sp. n. **A** dorsal side of head **B** distal part of leg III **C** leg III **D** female genital plate **E** anal valves. Scales: 0.1 mm (A &C), 0.01 mm (B, D & E)

Pseudocelli (pso) formula as 43/144/54464 dorsally and 11/000/00000 ventrally (Figs 3A, E); subcoxa 1 of legs I–III with 1 pso each (Fig. 4C). Parapseudocelli (psx) formula as 00/000/222401 ventrally and absent dorsally (Figs 3A, E); subcoxa 1 of legs I–III with 1 psx each (Fig. 4C). Pseudopore (psp) formula as 00/011/111100 dorsally and 00/111/000x00 ventrally (Figs 3A, E).

S-chaetae cylindrical, well differentiated, formula as 11/011/222111 dorsally and 11/000/000100 ventrally (Figs 3A, E); subcoxae 2 of legs I, II and III with 0, 0 and 1 S-chaeta respectively (Fig. 4C). Two posterior S-chaetae (Sp) present on head. S-microchaetae tiny and blunt, present on Th. II–III (Fig. 3A).

Head. Antennae short and distinctly segmented, as long as head. Length ratio of antennal segments I: II: III: IV = 1: 2: 2: 2. Ant. IV with two distinct thickened S-chaetae, subapical organite with apex globular and basolateral ms just above posterior chaetae (Fig. 3C). Ant. III sensory organ composed of 5 papillae, 5 guard chaetae, 2 small sensory rods, 2 granulated sensory clubs, the outer about twice as large as the inner, and a lateral ms (Fig. 3B). Ant. II with 15 chaetae. Ant. I with 9 chaetae. Antennal base with distinct granulation. PAO located on cuticular furrow built with a 3 lobed vesicle (Fig. 4A). Dorsal cephalic chaeta d0 absent, 3+3 p-chaetae between posterior a-pso on head (Fig. 4A). Mandible with strong molar plate and 4 apical teeth. Maxilla bearing 3 teeth and 6 lamellae. Maxillary palp simple with 1 basal chaeta and 2 sublobal hairs. Labral chaetae formula 4/342. Labium with 6 proximal, 4 basomedian (E, F, G, f) and 6 basolateral (a, b, c, d, e, e’) chaetae; labial type AC, papillae A–E respectively with 1, 4, 0, 3, and 2 guard chaetae (Fig. 3D). Postlabial chaetae 4+4 along ventral groove.

Body chaetotaxy. Ordinary chaetae differentiated in meso- and macrochaetae. Th. I tergum with 6+6 chaetae dorsally (Fig. 3A). Th. II–III and Abd. I–III terga with three chaetae on both side of axial line and no dorsal axial chaetae. Abd. IV tergum with one dorsal axial chaeta (m0), Abd. V without dorsal axial chaetae, Abd. VI with two axial chaetae (a0 and p0) (Fig. 3A). Th. I, II and III sterna without chaetae.

Appendages. Subcoxa 1 of legs I–III with 4, 5 and 5 chaetae, subcoxa 2 with 1, 4 and 4 chaetae respectively. Tibiotarsi of legs I, II and III with 20 (1, 8, 11), 20 (1, 8, 11) and 19 (1, 7, 11) chaetae (Fig. 4B). Unguis without inner teeth, with lateral teeth. Unguiculus slender and pointed, 0.6 times as long as inner edge of unguis, with inner basal lamella (Fig. 4B). Ventral tube with 6–7+6–7 distal chaetae and 2+2 basal chaetae, without anterior chaetae. Furca reduced to a small cuticular fold with 2+2 dental chaetae in two rows posteriorly; two manubrial rows of chaetae present (Fig. 3E).

Female genital plate with 17 chaetae (Fig. 4D), male genital plate with 19–24 chaetae. Anal valves with numerous acuminate chaetae; each lateral valve with a0 and 2a1; upper valves with chaetae a0, 2b1, 2b2, c0, 2c1, 2c2 (Fig. 4E). Anal spines present on indistinct papillae, 0.75 times as long as inner edge of unguis (Fig. 3A).

#### Etymology.

Named for the similarity with the Korean species *Oligaphorura montana* Weiner, 1994.

#### Ecology.

Found in coniferous forest.

#### Remarks.

The new species is very similar to the species *montana* collected in the mountain of North Korea, sharing the following characters: an increased number of pseudocelli dorsally; well marked base of antenna with 1 pseudocellus and 3 dorsal pseudocelli outside; subcoxa 1 of legs I–III with 1 pseudocellus each; dorsally S-chaetae formula as 11/011/22211 from head to Abd. V; S-microchaetae present on Th. II–III; head with chaeta p1 level with p2; Th. I, II and III sterna without chaetae; anal spines 0.75 times as long as inner edge of unguis; claw without inner teeth but with one lateral tooth; unguiculus with basal lamella. But they can be separated easily by the number of pseudocelli on Abd. V and VI terga (5 and 3 in *montana* versus 6 and 4 in *pseudomontana* sp. n.), parapseudocelli on the body (indistinct in *montana*, versus 00/000/222401 by half-sternite in *pseudomontana* sp. n.), the number of chaetae on Th. I tergum (7+7 in *montana* versus 6+6 in *pseudomontana* sp. n.), and the number of chaetae on tibiotarsi (19, 19, 18 in *montana* versus 20, 20, 19 in *pseudomontana* sp. n.).

## Supplementary Material

XML Treatment for Micraphorura changbaiensis

XML Treatment for Oligaphorura pseudomontana
